# Higher Preoperative Serum Neuropeptide Y Concentration May Be Associated with a Better Prognosis After Surgery for Colorectal Cancer

**DOI:** 10.3390/nu16223825

**Published:** 2024-11-07

**Authors:** Jacek Budzyński, Damian Czarnecki, Marcin Ziółkowski, Beata Szukay, Natalia Mysiak, Agata Staniewska, Małgorzata Michalska, Ewa Żekanowska, Krzysztof Tojek

**Affiliations:** 1 Department of Vascular and Internal Diseases, Nicolaus Copernicus University in Toruń, Ludwik Rydygier Collegium Medicum in Bydgoszcz, 85-168 Bydgoszcz, Poland; beata.szukay@cm.umk.pl (B.S.); natalia.mysiak1@wp.pl (N.M.); agata.stan1@op.pl (A.S.); 2 Department of Preventive Nursing, Nicolaus Copernicus University in Toruń, Ludwik Rydygier Collegium Medicum in Bydgoszcz, 85-067 Bydgoszcz, Poland; czarneckidamian@cm.umk.pl (D.C.); marcin.ziolkowski@cm.umk.pl (M.Z.); 3 Department of Pathophysiology, Nicolaus Copernicus University in Toruń, Ludwik Rydygier Collegium Medicum in Bydgoszcz, 85-094 Bydgoszcz, Poland; mmichalska@cm.umk.pl (M.M.); zorba@cm.umk.pl (E.Ż.); 4 Department of General and Minimally Invasive Surgery, Nicolaus Copernicus University in Toruń, Ludwik Rydygier Collegium Medicum in Bydgoszcz, 85-168 Bydgoszcz, Poland; krzysztof.tojek@cm.umk.pl

**Keywords:** neuropeptide Y, colorectal cancer, surgery, nutritional status, nutritional risk, prognosis

## Abstract

Background: The early identification of patients at risk of peri-procedural complications and poor prognosis is particularly important. We conducted our study to determine whether serum orexigenic neuropeptide Y (NPY) concentration is associated with nutritional status and prognosis among patients undergoing surgery for colorectal cancer (CRC). Materials and Methods: A cohort study with a 3-month follow-up was conducted with 84 consecutive inpatients who underwent elective surgery in one center between 2016 and 2019 for primary CRC. The clinical characteristics and nutritional status of all patients were assessed. In long-term follow-ups (median; IQR: 1322; 930–1788 days; average 3.6 years), the patients’ survival status was also checked during a telephone consultation. Results: Before CRC surgery, patients with serum NPY concentrations equal to or higher than the median value (661.70 pg/mL) had higher scores in their Mini Nutritional Assessment, Barthel, and Instrumental Activities of Daily Living (IADL) questionnaires, greater handgrip strength, a lower score in the Patient-Generated Subjective Global Assessment, and almost a three-times lower risk of perioperative complications, as well as higher Barthel and IADL scores and larger calf circumference at the 3-month follow-up visit in comparison to individuals with lower serum NPY concentrations. A higher serum NPY concentration was predictive of a low Nutritional Risk Screening 2002 score at the 3-month visit, and this was also found to have significantly influenced the patients’ survival during the 1200 days after CRC surgery. Conclusions: A higher preoperative serum NPY concentration may be related to lower nutritional risk, more favorable patient nutritional and functional status, and better survival, but further studies are required.

## 1. Introduction

Sporadic colorectal cancer (CRC) is one of the most prevalent and deadliest malignancies in the world. This neoplasm is also recognized as a diet- and obesity-related disease [1], which suggests, from one perspective, that appetite regulation and food intake are associated with carcinogenesis processes and, from another, that nutritional status plays a role in CRC patients’ prognoses. Specifically, about 10–20% of patients with cancer die due to undernutrition rather than the cancer itself [2,3], and malnutrition and sarcopenia have been found to be associated with a poor response to oncological treatment [4]. Therefore, the regulation of appetite and food intake in patients with CRC is particularly important. One of the organ systems that play a role in appetite regulation is the nervous system. Neuropeptides secreted from the central and peripheral systems, which include the visceral nervous system, exhibit both anorexic (e.g., leptin) and orexigenic (e.g., orexin, neuropeptide Y [NPY], and ghrelin) effects. Moreover, these neuropeptides may also take part in the molecular mechanism of cancer development through epigenetic, genetic (gene methylation), endocrine, paracrine, autocrine, pro-oxidative, and pro-inflammatory pathways, as well as in the progression of cancer diseases through metastasis formation [1,5,6,7]. These observations are supported by the significant associations found between CRC prevalence and the intake of some nutrients and nutraceuticals potentially involved in the molecular mechanisms of cancerogenesis, as well as the presence of several nutrigenomic polymorphisms, namely, those involved in folate metabolism (e.g., methylenetetrahydrofolate reductase produced by MTHFR), lipid metabolism (e.g., apolipoprotein A-I [ApoA-I]), oxidative stress (e.g., catalase), and inflammatory (e.g., tumor necrosis factor alpha [TNF-alpha]) responses [1].

We chose to study NPY as a potential prognostic biomarker in patients undergoing surgery due to CRC with the intention of healing for the following reasons: (a) NPY may stimulate appetite, and nutritional status is an important prognostic factor in cancer patients; (b) NPY may be involved in several homeostatic effects (e.g., energy balance, insulin resistance, emotional regulation, and alcohol dependence) that are indirectly related to CRC prevalence; (c) NPY may favor tumor growth, migration, and metastases; (d) NPY is recognized as a potential target in the treatment of cancer diseases; and (e) studies on the role of preoperative serum NPY concentration as a biomarker of CRC development, growth, differentiation, migration, and clinical progression in humans are few in number. NPY belongs to the pancreatic polypeptide family (which also includes peptides YY and PP), and it is widely expressed in the central and peripheral nervous systems [7,8,9,10,11,12,13,14,15]. NPY secretion and blood concentration can be stimulated by brain-derived neurotrophic factor (BDNF), orexigenic ghrelin, repeated stress, acute increases in insulin levels, and a high-fat and high-sugar diet; conversely, it is reduced by the anorexigenic leptin and peptide YY, as well as cytokines, such as interleukin-1ß and TNF-α. Recent studies have shown that NPY regulates angiogenesis through paracrine activity [7,15] and stimulates inflammatory-induced tumorigenesis through enhanced cell proliferation and the downregulation of micro-RNA-375 (miR-375) [5,16,17,18,19]. It has also been shown that hypermethylated NPY circulating tumor DNA (meth-ctDNA) may be useful as a biomarker of CRC metastasis and progression and a potential indicator for last-line treatment with regorafenib in metastatic CRC patients [6,10,15,20,21,22,23]. In CRC patients, progression-free survival has been found to be significantly shorter in patients with NPY DNA methylation compared to individuals without NPY gene methylation [10,22]. Serum NPY concentration has been found to be lower in CRC patients, and a decrease in this serum concentration has been associated with tumor size (>5 cm) and body weight loss (>3 kg) [22]. Contrary to these observations, NPY gene expression has been found to be higher in tumor tissue, independently of the tumor stage, compared to normal tissue [24]. However, it should be stated that NPY gene activity has been found to be associated with the reduced invasiveness potential of tumor cells in a concentration-dependent manner [25].

In light of the above-presented knowledge gaps and discrepancies, we undertook our study to determine whether there were associations between the serum NPY concentration and nutritional status assessment, nutritional risk, and prognosis among patients undergoing surgery for CRC with the intention of healing.

## 2. Patients and Methods

### 2.1. Patients

This prospective study involved 84 consecutive inpatients at our university hospital who underwent elective surgery due to primary CRC with curative intention between 2016 and 2019. The exclusion criteria were as follows: a need for emergency surgery (e.g., due to hemorrhage into digestive tract or bowel obstruction) and a lack of informed consent to participate in the study. Oral nutritional supplements, immunonutrition, and preoperative multimodal prehabilitation were not recommended before CRC surgery.

Before surgery, for each of the patients enrolled in this study, their medical history was obtained, and a physical examination was performed. An assessment of the anthropometric parameters of nutritional status and body composition (described in the subsection below) was performed for every patient. We also collected data concerning tumor size, clinical and histopathological stages, and perioperative complications, which were graded according to the Clavien–Dindo Classification [26]. All of the examinations performed during the baseline visit were repeated (though not all are presented in detail) at the 3-month control visit after discharge. The patients’ survival status and oncological treatment to date were checked during a telephone consultation after an average (median; IQR) of 1322 days and within a range of 930–1788 days (average 3.6 years).

### 2.2. Parameters of Nutritional Status and Body Composition Assessment

The following parameters of the nutritional and anthropometric status assessments were measured at baseline and at the 3-month follow-up visit: Patient-Generated Subjective Global Assessment (PG-SGA) (a score of 5 or more points was established as the cut-off value for a risk of malnutrition-related complications); Nutritional Risk Screening 2002 (NRS-2002) (a score of 3 or more points was assumed as the cut-off for a risk of malnutrition-related complications); Mini Nutritional Assessment (MNA) (a score of <17 indicates malnutrition, and a score in the range of 17–23.5 indicates a risk of malnutrition); body weight (kg); height (cm); body mass index (BMI) (kg/m^2^) (measured as the ratio of body weight to the square of height [m]); waist circumference (WC) (cm); waist-to-height ratio (WHtR) (measured as the ratio of WC [cm] to height [cm]); waist-to-hip ratio (WHR); mid-arm circumference (MAC) (cm); calf circumference (cm); and subscapular skinfold thickness (SST) (mm) and triceps skinfold thickness (TSF) (mm) (measured using a Harpenden MG-4800 manual skinfold caliper; BATY, Burgess Hill, UK). The handgrip strength (HGS) (kg) of the dominant hand was measured using an electronic dynamometer (Kern, Germany). We assumed the following cut-off values for a normal HGS: 16 kg for females and 27 kg for males [27].

Body composition was measured using whole-body bioelectrical impedance analysis (BIA) with a TANITA BC 420 MA device (TANITA Corporation, Tokyo, Japan). The following BIA parameters were analyzed: fat mass (FM) (expressed as a percentage of the total body weight [FM%] and as the absolute mass in kg [FM, kg]); fat-free mass (FFM) (kg); the visceral adipose tissue (VAT) score (a score of >12 was the established cut-off for abdominal adiposity diagnosis); and skeletal muscle mass (SMM) (expressed as a percentage of the total body mass [SMM%] and as the absolute mass in kg [SMM, kg]) [28]. The following established sarcopenia criteria were used for BIA: <37% for males and <31.5% for females [27]. Moreover, because abdominal computed tomography (CT, Revolution HD, GE Healthcare, Buckinghamshire, UK) was performed on every CRC patient before surgery (range of slice thickness: 1–5 mm), the cross-sectional areas (CSAs) of the skeletal muscle (SM) (attenuation limit −30 to 150 Hounsfield units [HU]), VAT (attenuation limit −150 to −50 HU), and subcutaneous adipose tissue (SAT) at the third lumbar (L3) vertebra were manually selected as specific regions of interest (ROIs) using OsiriX software (Pixmeo SARL, Bernex, Switzerland, version: UDI-PI: 14.1.0) [29].

Functional status was also established using, for example, Barthel, activities of daily living (ADL), and Instrumental Activities of Daily Living (IADL) indices.

### 2.3. Biochemical Determinations

Blood sampling from the ulnar vein of each patient was performed at admission, between 7 am and 8 am, on an empty stomach. The laboratory analyses were performed in the hospital’s diagnostic laboratory using standard methods, and they were as follows: blood morphology with a detailed determination of white blood cell distribution and total lymphocyte count [TLC]; glucose; albumin; C-reactive protein; and carcinoembryonic antigen (CEA). The preoperative Onodera Prognostic Nutritional Index (OPNI) was also calculated according to the following formula: [10 × blood albumin concentration (g/L)] + [0.005 × TLC (g/L)] [30].

The serum NPY concentration was assessed preoperatively using an immunoenzymatic ELISA kit from Cloud-Clone Corp. (cat. no. CEA607Hu, Katy, TX, USA) in accordance with the manufacturer’s instructions. The serum samples were centrifuged at a temperature of 4 °C and a spin speed of 3500 revolutions per minute. The serum samples were then stored at a temperature of −80 °C until analysis. We analyzed only one neuropeptide due to limited financial support.

### 2.4. Outcomes Measured

The relationships between the serum NPY concentration before surgery and the following variables were assessed: the scores of nutritional and functional status questionnaires; the values of the anthropometric parameters of the nutritional status assessment; the BIA parameters of body composition obtained at baseline and at the 3-month visit, as well as the SM, SAT, and VAT CSA CT scans at L3; the length of in-hospital stay (LOS); the occurrence of perioperative complications, graded according to the Clavien–Dindo Classification [26]; and cancer advancement (WHO clinical stages I–IV). Patients’ survival and history of oncological treatment after discharge were checked via a telephone consultation with patients or with a patient’s family member (when a patient died) after a median follow-up period of 1322 days (IQR: 930–1788 days; average: 3.6 years).

#### 2.4.1. Bioethics

The investigation was conducted in compliance with the guidelines detailed in the Declaration of Helsinki for medical research after receiving permission from the Bioethics Committee of the Nicolaus Copernicus University in Toruń functioning at Collegium Medicum in Bydgoszcz (No. KB 595/2015, approval date: 29 September 2015).

#### 2.4.2. Statistics

Statistical analysis was conducted using the licensed version of the statistical software Statistica, version 13.3, developed by Tibco Software, Inc., Palo Alto, CA, USA, (2017). The normal distribution of the study variables was checked using the Kolmogorov–Smirnov test. Depending on the type of variable distribution, the results are presented as the median; the interquartile range (IQR); the mean ± standard deviation; or *n*, %. In addition, the statistical significance of the differences between groups was verified using the Mann–Whitney U non-parametric test, Student’s *t*-test, or Chi^2^ test. The statistical significance level was set at a *p*-value of <0.05. A Kaplan–Meier curve was determined in the survival analysis.

Assuming a 10%, 20%, and 30% difference in patients’ survival at *p* < 0.05 and 80% statistical power for the long-rank test in the survival analysis, the minimum sample size in one group was 204, 67, and 37, respectively. To achieve statistical significance regarding the average 10% differences between mean values in a comparison of the study subgroups (e.g., serum NPY concentration lower and higher or equal to the median value) using Student’s *t*-test with the same statistical assumptions (*p*-value, statistical power), the minimum sample required in one group was 17 or 64, depending on the value of the standard deviation (10% or 20% of the mean value, respectively).

## 3. Results

### 3.1. Clinical Characteristics in Relation to the Median NPY Value

Before CRC surgery, in comparison to individuals with a lower preoperative serum NPY concentration, patients with a serum NPY concentration higher than or equal to the median value (median; IQR: 661.70; 375.7–913.0 pg/mL) had slightly, but not clinically significantly, higher scores in the MNA, Barthel, and IADL questionnaires; were more likely to have an HGS greater than the cut-off value established for a sarcopenia diagnosis; and had (with borderline statistical significance) an SM CSA (via CT) of ≥ 92 cm^2^ at L3 (Table 1). Moreover, at the 3-month visit, patients with higher serum NPY concentrations also had higher Barthel and IADL scores and a greater calf circumference than their counterparts, but the differences were not clinically significant (Table 1). Patients with a higher serum NPY concentration also had lower scores in the PG-SGA survey before surgery and a lower mean percentage loss of body weight during the 3 months before surgery (Table 1). In addition, they were almost three times less likely to suffer from all-cause (both surgical and non-surgical) perioperative complications (Table 2) and have an NRS-2002 of ≥3 at the 3-month visit than their counterparts (Table 1). However, we did not find statistically significant differences between the groups with regard to demographic data; clinical factors; some scores in the nutritional risk questionnaires (e.g., NRS-2002); the anthropometric parameters of the nutritional status assessment; the parameters of body composition in BIA; the values for SM, VAT, and SAT CSA via CT; biochemical parameters; or the clinical stages and grading of CRC (not all of the data are presented in detail). To avoid presenting excessive data, we chose only those data obtained at the 3-month visit that statistically significantly differed between the groups (Table 1). All of the parameters which were lower/higher preoperatively in the group with a low concentration of NPY were also lower/higher at the 3-month visit.

### 3.2. Prognostic Value of Preoperative NPY (Receiver Operating Characteristic [ROC] Analysis)

**Figure 1:**
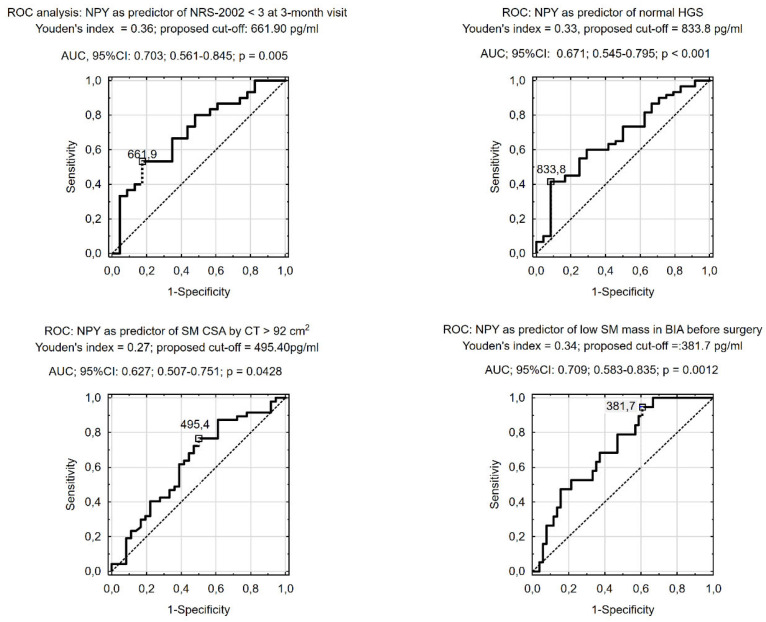
Receiver operating characteristic (ROC) analysis of the preoperative neuropeptide Y (NPY) cut-off value for predicting patients without an increased nutritional risk at a 3-month follow-up visit.

**Figure 2:**
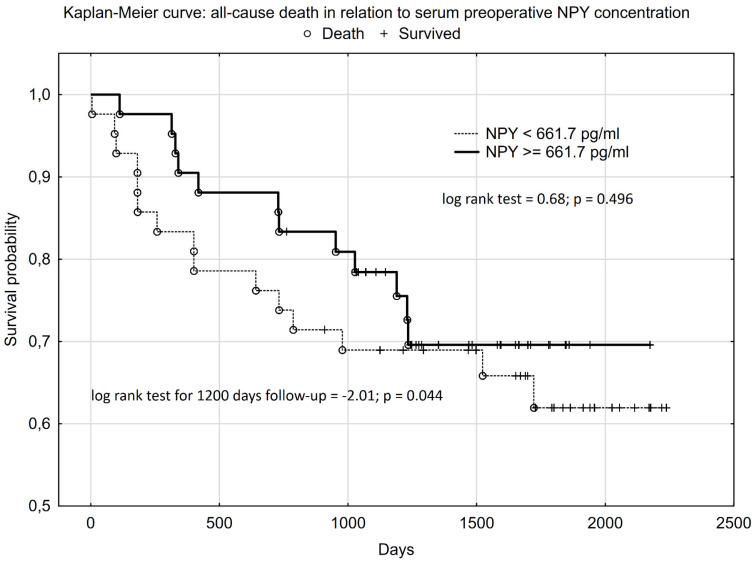
The Kaplan–Meier survival probability among CRC patients in relation to the preoperative serum neuropeptide Y (NPY) concentration.

ROC analysis indicated that preoperative serum NPY concentration was predictive of having normal HGS before surgery, an SM CSA of ≥92 cm^2^ in CT slides at L3, and a sarcopenia diagnosis according to the BIA criteria (i.e., percentage of SM < 37% in males and < 31.5% in females). We also found that a low preoperative serum NPY concentration before CRC surgery was predictive of increased nutritional risk at the 3-month follow-up visit (Figure 1). However, we did not observe the NPY cut-off predicting all-cause death during follow-up in the ROC analysis. Therefore, in the Kaplan–Meier analysis, we used the median of the preoperative serum NPY concentration (661.7 pg/mL) to compare the patients’ survival during an average of 3.6 years of follow-up. It should be underlined that the median NPY value used was similar to the cut-off for preoperative NPY concentration obtained in the ROC analysis, which was predictive of increased nutritional risk (NRS_2002 ≥ 3) at the 3-month visit (Figure 1). A higher preoperative NPY serum concentration had a statistically significant influence on the patients’ survival up to 1200 days after CRC surgery (log-rank test = −2.01; *p* = 0.044) in the Kaplan–Meier analysis. However, when we performed the analysis for the entire follow-up (average of 3.6 years), the Kaplan–Meier curves overlapped, and the statistically significant difference disappeared (log-rank test = 0.68; *p* = 0.496) (Figure 2). However, the power of the survival analysis performed only amounted to 0.228 due to the small sample size and small number of outcomes measured. None of the CRC patients enrolled in this study required additional surgery, chemotherapy, or radiotherapy during the follow-up period beyond those presented in Table 2.

## 4. Discussion

One of the significant factors influencing the prognosis of CRC patients undergoing surgery is their nutritional status and risk, as well as their appetite and food intake. The early identification of patients threatened by death or by a worsening of nutritional risk and status after CRC surgery is particularly important because it offers the possibility of introducing preventive action, for example, through personalized nutritional support. Therefore, the most important observation to emerge from our study is that patients with higher preoperative serum NPY concentration, which is an orexigenic neuropeptide also favoring cancer growth and migration, had a greater probability of survival during the 1200 days after CRC surgery performed with curative intention (Figure 2). Unfortunately, the statistical power of this survival analysis was low (0.228). We also found that a lower preoperative serum NPY concentration (<661.7 pg/mL) could help to identify patients with a higher percentage of weight loss before surgery (Table 1), an increased nutritional risk (NRS-2002 score ≥ 3), and a slight deterioration in their functional status at the 3-month visit after CRC surgery (Table 1, Figure 1), probably due to decreased appetite and food intake. Moreover, we also revealed that, compared to patients with a lower serum NPY concentration (<661.7 pg/mL), those with a higher serum NPY concentration, despite having a similar advancement of CRC, also had slightly more favorable scores in the validated nutrition screening and assessment tools (MNA and PG-SGA) (Table 1); a three times lower likelihood of perioperative complications (mainly in Grades I and II of the Clavien–Dindo Classification (Table 2)); a slightly better functional status (Barthel and IADL scales) both before surgery and at the 3-month visit after CRC surgery; and higher SMM indices before surgery (e.g., an SM CSA of ≥ 92 cm^2^ at L3) and at the 3-month visit (calf circumference) (Table 1).

To the best of our knowledge, our report is the first to describe the observation that better survival, a lower likelihood of perioperative complications, and more favorable nutritional (NRS-2002 score < 3, Figure 1) and functional outcomes are associated with higher preoperative serum NPY concentrations in patients after CRC surgery. These favorable associations can be explained by the orexigenic activity of NPY [1,5,6,7,8,9,10,31] and the maintenance of NPY-dependent appetite and food intake in response to an increase in pre- and postoperative catabolism and energy demand. Such an explanation of the obtained results also shows that orexigenic NPY activities leading to higher appetite and food intake and lower nutritional risk (NRS-2002) at the 3-month visit (Table 1, Figure 1) surpassed the potential NPY properties favoring cancer growth and migration [5,7,15,16,17,18,19] during the follow-ups that were conducted up to 1200 days after CRC surgery (Figure 2). The statement that NPY activities regulating a patient’s nutritional risk and status have greater prognostic importance than those favoring cancer growth and migration is also supported by the observation that similar NPY cut-off values were predictive of both increased nutritional risk at the 3-month visit (<661.9 pg/mL, Figure 1) and worse survival during the average 3.6 years of follow-up (<661.7 pg/mL; Figure 2).

The clinical importance of our observation is that identifying patients with a lower preoperative serum NPY concentration (<661.7 pg/mL) is likely to be useful as a biomarker in the early identification of patients with a lower survival probability, an increased risk of perioperative complications (Table 2), a greater nutritional risk (3 months after surgery), and a greater likelihood of unplanned weight loss, postoperative sarcopenia (lower calf circumference at 3-month visit, Table 1), lower HGS, and deterioration in everyday functioning (e.g., IADL score). The association of the unfavorable prognosis of patients after CRC surgery with abnormal scores in nutritional risk scales (e.g., NRS-2002 score ≥ 3 or a low Geriatric Nutritional Risk Index) and a small calf circumference (Table 1) has been previously reported [29,30,31,32,33,34,35]. These patients could potentially benefit from nutritional support and/or appetite improvement (e.g., thorough orexigenic substance supplementation) after surgery for CRC. Evidence was previously found that nutritional support using oral nutritional supplements improves patients’ prognoses and prevents postoperative weight loss, BMI reduction, and postoperative sarcopenia when applied both before surgery as multimodal prehabilitation [36] and after CRC surgery [37,38].

## 5. Study Limitations and Strengths

As with the majority of studies, our analysis has some limitations that may reduce the strength of the conclusions obtained. The small number of patients included in our study (*n* = 84) and the one-center study design should be considered the main limitations. The small number of CRC patients in the study sample was related to the low statistical power of the survival analysis and makes it impossible to perform, for example, multifactorial survival analysis (e.g., Cox’s proportional hazard regression model), which would help to identify factors influencing, in addition to preoperative serum NPY concentration, the deterioration of a patient’s nutritional status and risk, as well as a patient’s survival. We also did not monitor food and diet supplement (e.g., proteins, omega-3 fatty acids, hydroxy metylbutyrate [HMB], and vitamin D) intake and appetite preoperatively or after a patient’s discharge. In addition, we did not confirm the association between the preoperative serum NPY concentration and CRC advancement. Moreover, we did not perform serial measurements of the serum NPY concentrations, and as stated above, a CRC patient’s survival after surgery is known to depend on several factors, and single preoperative NPY measurements might be biased. Furthermore, our study was terminated prematurely due to a failure in the device used for the body composition analysis and a change in the surgical clinic, meaning all oncological patients were referred to the oncological center in our city. This change to the oncological center was also the reason that relatively few patients are operated on per year at our center, and so not many patients were enrolled in this study. Nevertheless, the novelty of this study is unquestionably a strong point: to the best of our knowledge, it is the first study worldwide to assess the role of preoperative serum NPY concentration in humans. In addition, in this study, we revealed the predictive role of higher preoperative serum NPY concentrations in the identification of patients with a greater likelihood of survival, an almost three-times lower risk of perioperative complications, and a lower nutritional risk (NRS-2002 score < 3) at the 3-month follow-up visit after surgery for CRC performed with curative intention. Nevertheless, our observations need to be confirmed in large-sample and well-powered studies.

## 6. Conclusions

A higher preoperative serum NPY concentration may be related to a greater likelihood of CRC patients’ survival over the 1200 days after CRC surgery, an almost three-times lower risk of perioperative complications, and lower nutritional risk and slightly better functional status 3 months after the operation. These observations show the greater role that NPY’s orexigenic properties have compared to those favoring cancer development, growth, and progression in patients who have undergone CRC surgery with curative intention. Further studies with greater statistical power are needed to confirm our observations.

## Figures and Tables

**Table 1:** A comparison of the clinical characteristics between patients with a preoperative serum NPY concentration equal to, greater than, or lower than the median value (661.70 pg/mL).

Parameter	Preoperative	Preoperative	*p*
	NPY ≥ 661.70 pg/mL	NPY < 661.70 pg/mL	
	(*n* = 42)	(*n* = 42)	
Results obtained preoperatively			
Preoperative serum NPY (pg/mL)	937.84 ± 212.14	367.25 ± 176.49	<0.001
Age (years)	66.71 ± 11.03	67.95 ± 12.23	0.627
Male gender (*n*, %)	28 (66.7)	22 (52.4)	0.187
NRS-2002 (score)	2.79 ± 0.84	3.02 ± 1.00	0.241
NRS-2002 score ≥ 3	24 (57.1)	27 (64.3)	0.503
MNA (score)	26.26 ± 2.58	24.38 ± 3.16	0.004
PG-SGA (score)	4.17 ± 2.56	6.21 ± 5.40	0.029
GNRI (score)	105.95 ± 5.40	103.07 ± 9.05	0.081
OPNI (score)	42.34 ± 3.55	40.78 ± 5.78	0.138
Barthel (score)	99.64 ± 2.39	97.62 ± 5.09	0.021
ADL (score)	6.00 ± 0.00	5.93 ± 0.26	0.079
IADL (score)	23.67 ± 1.14	22.83 ± 2.19	0.031
Mean percentage loss of body weight before surgery during the 3 months before surgery (%)	−2.79 ± 6.00	−7.03 ± 7.66	0.014
BMI (kg/m^2^)	29.67 ± 12.20	29.31 ± 12.59	0.893
Waist circumference (cm)	100.57 ± 12.14	99.88 ± 15.18	0.819
Waist-to-hip ratio	0.97 ± 0.10	1.20 ± 1.49	0.323
Waist-to-height ratio	0.59 ± 0.07	0.60 ± 0.09	0.771
Mid-arm circumference (cm)	28.66 ± 3.44	28.74 ± 3.56	0.920
Calf circumference (cm)	37.32 ± 3.82	36.56 ± 3.43	0.343
HGS of dominant hand (kg)	33.82 ± 11.22	29.51 ± 13.48	0.115
Percentage of patients achieving HGS gender-specific cut-off value	35 (83.3)	25 (59.5)	0.016
Fat mass in BIA (%)	29.12 ± 8.84	29.50 ± 9.80	0.855
VAT (score)	12.45 ± 3.76	12.44 ± 5.84	0.992
Percentage of skeletal mass (%)	37.86 ± 5.92	39.05 ± 6.68	0.404
Blood albumin (g/L)	4.23 ± 0.36	4.08 ± 0.58	0.138
CRP (mg/L)	8.71 ± 14.03	9.81 ± 14.29	0.723
Hemoglobin (g/L)	12.72 ± 2.18	12.71 ± 1.89	0.987
Lymphocytes (G/L)	1.94 ± 0.63	2.07 ± 1.89	0.577
Total cholesterol (mg/dL)	182.60 ± 48.49	170.10 ± 42.09	0.211
Glucose (mg/dL)	110.71 ± 23.47	111.05 ± 18.91	0.943
Creatinine (mg/dL)	0.87 ± 0.23	0.86 ± 0.21	0.754
SM CSA by CT before surgery (cm^2^)	105.11 ± 28.49	94.91 ± 36.02	0.156
SAT CSA by CT before surgery (cm^2^)	174.30 ± 84.06	177.91 ± 77.51	0.840
SM CSA ≥ 92 cm^2^ before surgery (*n*, %)	28 (66.7)	19 (45.2)	0.061
SAT CSA ≥ 118 cm^2^ before surgery (*n*, %)	29 (69.0)	33 (78.6)	0.230
VAT CSA ≥ 241 cm^2^ before surgery (*n*, %)	8 (19.0)	6 (14.3)	0.591
Selected results obtained at 3-month visit			
NRS-2002 score ≥ 3 at 3-month visit	8 (19.0)	24 (57.1)	0.001
Barthel score at 3-month visit	100.00 ± 0.00	92.50 ± 17.71	0.022
ADL score at 3-month visit	6.00 ± 0.00	5.72 ± 1.06	0.149
IADL score at 3-month visit	23.94 ± 0.25	21.64 ± 3.75	0.001
HGS of dominant hand at 3-month visit (kg)	32.44 ± 9.97	27.32 ± 12.94	0.079
Calf circumference at 3-month visit (cm)	40.74 ± 6.02	37.56 ± 5.69	0.031

Abbreviations: ADL = activities of daily living; BIA = bioelectrical impedance analysis; BMI = body mass index; CRP = C-reactive protein; CSA = cross-sectional area; CT = computed tomography; GNRI = Geriatric Nutritional Risk Index; HGS = handgrip strength; IADL = Instrumental Activities of Daily Living; MNA = Mini Nutritional Assessment; NPY = neuropeptide Y; NRS-2002 = Nutritional Risk Screening 2002; OPNI = Onodera Prognostic Nutritional Index; PG-SGA = Patient-Generated Subjective Global Assessment; SAT = subcutaneous adipose tissue; SM = skeletal muscle; and VAT = visceral adipose tissue.

**Table 2:** Prevalence of the measured outcomes and the parameters of colorectal tumor size and clinical advancement in patients with a preoperative serum NPY concentration that is equal to, greater than, or lower than the median value (661.70 pg/mL).

Parameter	Preoperative	Preoperative	*p*
	NPY ≥ 661.70 pg/mL	NPY < 661.70 pg/mL	
	(*n* = 42)	(*n* = 42)	
Length of in-hospital stay (days)	8.88 ± 5.32	9.15 ± 4.81	0.809
All-cause death during follow-up (*n*, %)	12 (28.6)	15 (35.7)	0.483
Length of follow-up (days)	1265.95 ± 497.31	1326.05 ± 723.38	0.658
Carcinoembryonic antigen (ng/mL)	6.50 ± 10.5	4.58 ± 6.49	0.310
Tumor size (cm)	4.29 ± 1.88	4.73 ± 3.32	0.472
Localization (rectum/colon) (*n*, %)	15 (35.7)	19 (45.2)	0.344
	27 (64.3)	23 (54.8)	
Histopathological grade (G1/G2/G3) (*n*, %)	1 (2.4)	2 (4.8)	0.169
	39 (92.9)	35 (83.3)	
	2 (4.8)	5 (11.9)	
WHO CRC clinical advancement (stage I/II/III/IV) (*n*, %)	10 (23.8)	11 (26.2)	0.994
	11 (26.2)	11 (26.2)	
	17 (40.5)	16 (38.1)	
	4 (9.5)	4 (9.5)	
Astler–Coller classification (A/B/C/D) (*n*, %)	1 (2.4)	4 (9.5)	0.467
	19 (45.2)	21 (50.0)	
	18 (42.9)	14 (33.3)	
	4 (9.5)	3 (7.1)	
Lymph gland metastases (*n*, %)	18 (42.9)	17 (40.5)	0.565
All-cause perioperative complications (*n*, %)	5 (11.9)	13 (31.0)	0.049
CDC Grade I and II	5 (11.9)	9 (21.4)	0.242
CDC Grade IIIa	0	4 (9.5)	0.041
Adjuvant chemotherapy (*n*, %)	23 (54.8)	19 (45.2)	0.258
Neoadjuvant radiotherapy (*n*, %)	15 (35.7)	11 (26.2)	0.271

Abbreviations: CDC = Clavien–Dindo Classification; CRC = colorectal cancer; and NPY = neuropeptide Y.

## Data Availability

The data presented in this study are available on request from the corresponding authors due to privacy restrictions.
